# Tigecycline-induced coagulation gene prognostic prediction model and intestinal flora signature in AML

**DOI:** 10.3389/fimmu.2024.1486592

**Published:** 2024-11-14

**Authors:** Feng-Luan Zhong, Jia-Jun He, Kun-Hao Bai, Ruo-Nan Shao, Guo-Yan Wu, Xiao-Peng Tian, Da-Wei Wang, Yu-Jun Dai, Si-Liang Chen

**Affiliations:** ^1^ Department of Hematology, Peking University Shenzhen Hospital, Shenzhen, Guangdong, China; ^2^ State Key Laboratory of Oncology in South China, Guangdong Provincial Clinical Research Center for Cancer, Sun Yat-sen University Cancer Center, Guangzhou, China; ^3^ Department of Hematologic Oncology, Sun Yat-sen University Cancer Center, Guangzhou, Guangdong, China; ^4^ Department of Endoscopy, Sun Yat-sen University Cancer Center, Guangzhou, China; ^5^ Department of Critical Care Medicine, Shanghai General Hospital, Shanghai Jiao Tong University School of Medicine, Shanghai, China; ^6^ Jiangsu Institute of Hematology, The First Affiliated Hospital of Soochow University, Suzhou, Jiangsu, China; ^7^ National Research Center for Translational Medicine, Ruijin Hospital Affiliated to Shanghai Jiao Tong University School of Medicine, Shanghai, China

**Keywords:** tigecycline, intestinal microbiota, 16S sequencing, prognosis model, AML

## Abstract

Infection is among the most common causes of death in patients with acute myeloid leukemia (AML) after chemotherapy. The anti-tumor effect of the intestinal microbiota in patients with AML is increasingly being recognized. Tigecycline, a broad-spectrum antibiotics, plays a vital role in the anti-infection treatment of AML patients with neutropenia and accompanying infections. Previously, this group reported that long-term use of tigecycline caused coagulation dysfunction in patients with hematological malignancies, increasing the risk of casualties. RNA sequencing was performed on CHO cells before and after tigecycline treatment. Further, the combined analysis of AML prognostic differentially expressed genes revealed 13 genes affected by tigecycline and closely related to AML prognosis. These genes were used for modeling analysis, and the results showed that the prepared model significantly improved the prognostic prediction efficiency for AML patients. The model also explored the correlation between prognosis score and immune cells infiltrating tumors and immune therapy targets. Moreover, 16S sequencing was performed on fecal samples from AML patients before and after tigecycline treatment. The results revealed that tigecycline significantly altered the distribution of intestinal microbiota in AML patients - These changes in microbiota are related to chemotherapy resistance. This study emphasizes the importance of intestinal microbiota in AML prognosis. Thus, the findings of this study show that the long-term use of antibiotics can not only cause dysbiosis of the intestinal microbiota but also indirectly affect the sensitivity of chemotherapy drugs, affecting the prognosis of AML patients.

## Introduction

World Health Organization International Cancer Research Agency (IRAC) reported 19.29 million new cancer cases worldwide in 2020. Of these, 4.57 million new cancers were reported in China, accounting for about 23.7% of the total cases reported. Although chemotherapy can effectively extend the survival of patients with cancer (1), the medication scheme, dosage, and individual differences during chemotherapy cause varying degrees of bone marrow suppression in patients. Because chemotherapy plays a vital role in tumor treatment, the bone marrow suppression problem cannot be ignored (2, 3). According to relevant statistics, 80% of the patients will experience bone marrow suppression during tumor radiotherapy and chemotherapy, reducing neutral granulocytes, platelets, and red blood cells, leading to infection, bleeding, anemia, and other phenomena (4, 5). The decrease in neutral granulocytes is most prominent during bone marrow suppression. It decreases immunity and develops symptoms such as severe infection, fever, and fatigue. Infection is the most common and worst complication in elderly AL patients after chemotherapy and one of the causes of death (6).

Tigecycline can be quickly and widely distributed in the body to treat adult complex skin and soft tissue infections (CSSSIS) and adult complex abdominal infections (CIAIS) (7, 8). Tigecycline can induce apoptosis of leukemia (9). Inhibitory drugs combined with autophagy can further increase tigecycline’s specific anti-leukemia effect and reverse CML resistance (10). Moreover, tigecycline can inhibit cell vitality, block cell cycles, and induce cell autophagy to treat multiple myeloma (11). Although various adverse reactions are reported on the diameter application, coagulation dysfunction is a rare side effect (12). The coagulation dysfunctions are related to multiple factors when treating immunosuppressive AL patients (13).

Abnormal severe infection and coagulation function are common causes of death in patients with leukemia. In this study, a novel prognostic model was constructed by genes regulated by tigecycline in patients with AML. Further, the tumor immune microenvironment and the intestinal flora changes induced by tigecycline were also investigated.

## Materials and methods

### Patient samples

Eleven patients with hematological malignancies diagnosed at the Sun Yat-sen University Cancer Center between 2021 and 2022 were enrolled in this study. All participants provided written informed consent per the regulations of the Institutional Review Boards of the Hospitals in agreement with the Declaration of Helsinki.

### RNA-sequencing

The feature that CHO cells could be cultured in suspension and adherent at the same time made them be one of the important cells for platelet function research, and had been widely used in platelet function research in the world (14, 15). The CHO cells treated with tigecycline (0 mg/mL and 0.2 mg/mL) for 48 h were used for RNA sequencing, following previously reported methods (13). The raw data and proceed data were uploaded to NCBI (GSE198830). Here, the “edgeR” package was used to identify the differentially expressed genes (DEGs) between the CHO cell group treated with 0 mg/ml tigecycline and 0.2 mg/ml tigecycline. The following criteria were used: (a) fold change > 2 (Log_2_FC>1 or <-1); (b) false discovery rate (FDR) < 0.05; and (c) gene expression levels > 1.

### Quantitative real-time PCR

The expression of CRGs were measured by qRT-PCR. We followed the manufacturer’s guidelines using the ESscience assay (QP002) and employed the 2−ΔΔCt formula to examine the relative expression levels. Primers are listed in **Supplementary Table S1**.

### 16S rRNA gene sequencing

Eleven patients with hematological malignancies were recruited; their feces specimens were collected before and on the third day using tigecycline for microorganisms 16s sequencing. The community DNA fragments were sequenced via the Illumina platform using paired-end sequencing. Both DADA2 and Vsearch were used to sequence denoising. Greengenes database (Release 13.8, http://greengenes.secondgenome.com/) was used to annotate species taxonomy and construct the phylogenetic tree. Next, multiple advanced bioinformatics methods were applied to analyze data, such as species composition analysis, alpha diversity analysis, beta diversity analysis, species difference and marker species analysis, and association network analysis. The raw data and proceed data were uploaded to NCBI (No: PRJNA967642).

### Data acquisition

Gene expression information and the corresponding survival time data of patients with hematological malignancies were retrieved from public AML databases, including The-Cancer-Genome-Atlas (TCGA) and Gene-Expression-Omnibus (GEO: GSE37642, GSE71014, and GSE106291). Log_2_ transformation was performed to normalize the expression profiles (**Supplementary Figure S1**).

### Tigecycline-related prognostic signature model

The TCGA cohort was used as a training cohort, and other GEO databases were used as the validation cohorts (16). Overall survival (OS)-related DEGs of tigecycline were screened via Venn diagram analysis (17). The prognostic tigecycline signature was constructed using the LASSO regression analysis based on 10-fold cross-validation penalized maximum likelihood estimators. The minimum criterion was used to choose the optimal penalty parameter (λ) values with a repetition frequency 1000. The CRG risk score (RS) was calculated for each AML patient using the following formula: RS = (0.041 * SEMA3C expression level) + (0.04 * SRC expression level) + (0.015 * PYCARD expression level) + (0.012 * MFHAS1 expression level) + (0.011 * ST6GALNAC4 expression level) + (0.0005 * HMGA1 expression level) - (0.038 * PLD1 expression level). Patients were further assigned to high-risk or low-risk sets based on the median of the RS. Kaplan-Meier and time-dependent receptor operating characteristic (ROC) curves were used to assess the predictability of the tigecycline signature. The PRC curve was analyzed by PRROC package in R.

### Immune -infiltrating analysis

The relationship between the tigecycline signature and immune cells involved in the tumor microenvironment was evaluated using the Tumor Immune Estimation Resource (TIMER; cistrome.shinyapps.io/timer). The Pearson correlation analysis was done for an in-depth analysis of the relationship between Tregs and tigecycline signature. Also, the current critical immune checkpoints (ICKs), including PDCD1, CD274, TIGIT, CTLA4, LAG3, and IDO1, were examined to indirectly speculate the treatment response of immune checkpoint inhibitors in AML cells.

### Statistical analysis

SPSS statistical software was used for normalization analysis. Mean ± standard deviation was used to present the measured data, and t-test or corrected t-test was used to analyze the significance of tigecycline signature and other variances. The χ^2^ test was used to compare the categorical data. Continuous variables were subjected to analysis employing either the parametric Student’s t-test or the non-parametric Wilcoxon rank-sum test, depending on the normality and homogeneity of variance assumptions. The log-rank test was employed to assess differences in OS distributions between two cohorts. For the evaluation of diagnostic accuracy, the time-dependent ROC analysis package was implemented to construct ROC curves and ascertain the AUC metric. Prognostic factors were identified via univariate and multivariate Cox proportional hazards regression analyses, facilitating the estimation of independent prognostic indicators (**Supplementary Texts S1, S2**). *P* < 0.05 indicated a statistical difference, and *P* > 0.05 showed no statistical difference.

## Result

### Prognostic significance of tigecycline-related genes in AML

**Figure 1:**
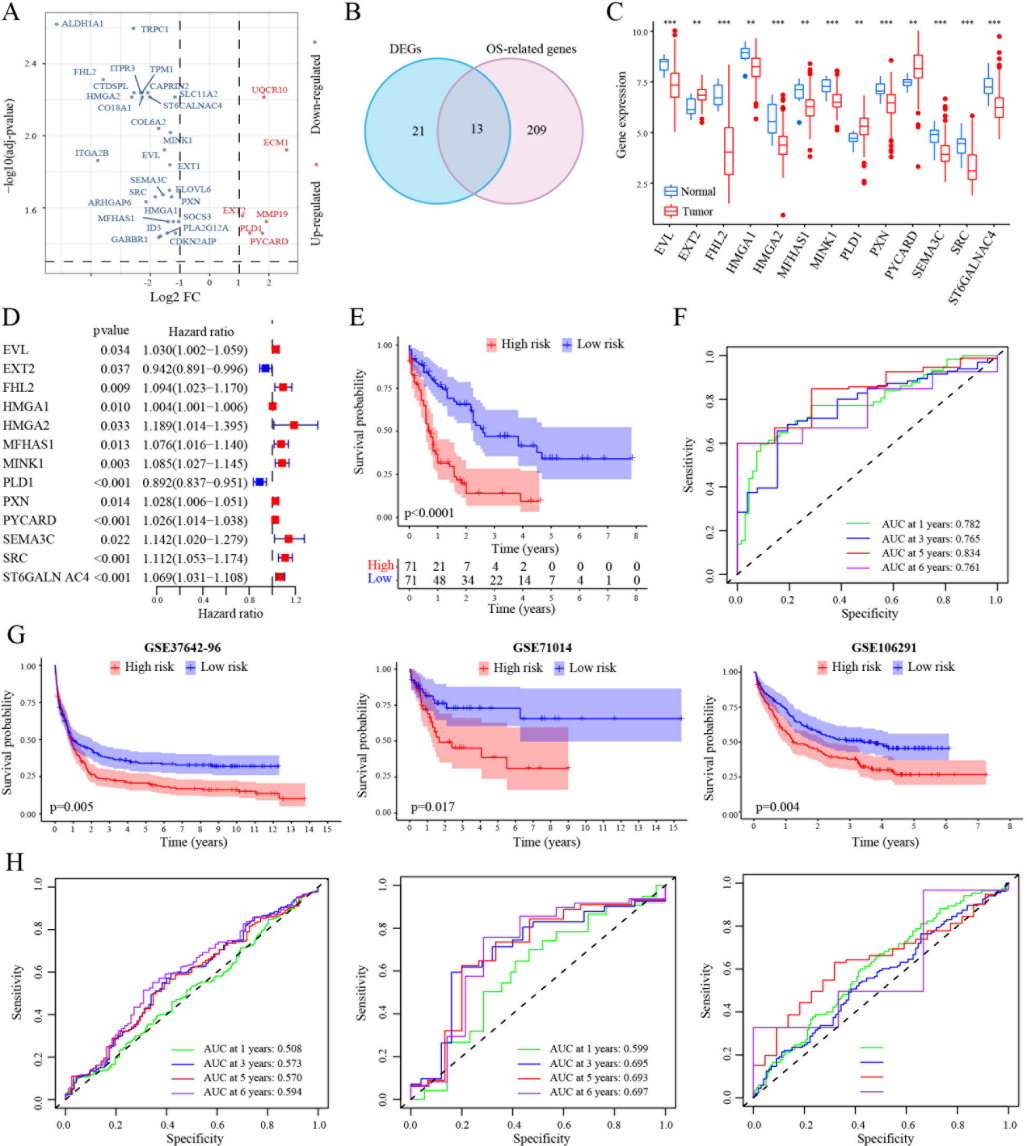
Construction of a prognostic signature in the TCGA-LAML cohort. **(A)** Volcano plot exhibiting 34 DEGs in GSE1159 cohort. **(B)** Venn diagram to identify 13 overlapping prognostic DEGs. **(C)** Expression signatures of 13 prognostic DEGs in GSE1159 cohort. **(D)** Forest plots showing the results of the univariate Cox analysis between gene expression and OS in TCGA-LAML cohort. **(E)** Kaplan-Meier curve of 7 MRG profile in the TCGA-LAML cohort. **(F)** Time-dependent ROC analysis of 7 MRG profile in the TCGA-LAML cohort. **(G, H)** Kaplan-Meier curve and time-dependent ROC analysis of 7 MRG profile in the GSE37642-96 (n=417), GSE71014 (n=104) and GSE106291 (n=250) cohorts, respectively. **, p < 0.01; ***, p < 0.001.

RNA sequencing of CHO cells was conducted before and after tigecycline (0.2 mg/ml) treatment. The differentially expressed genes between these two groups were identified using the “edgeR” package (**Supplementary Figures S2A-C**). We first identified the gene expression differences in CHO cells before and after treatment with tigecycline through RNA sequencing. The results showed that the expression of most genes was downregulated after treatment with tigecycline (**Figure 1A**). We further validated using AML cell lines and found that the changes in these genes in AML cell lines treated with tigecycline were highly consistent with the changes in CHO cells (**Supplementary Figure S9**). Subsequently, these hamster derived genes were compared with human derived genes. Then, the TCGA database was used to analyze the prognostic significance and expression differences of the transformed human genes. Thirteen different genes were regulated by tigecycline, which was also closely related to AML prognosis (**Figure 1B**). Of these, ten genes (*EVL, FHL2, HMGA1, HMGA2, MFHAS1, MINK1, PXN, SEMA3C, SRC*, and *ST6GALN AC4*) showed lower expression in AML cells, and the remaining three genes (EXT2, PLD1, and PYCARD) showed elevated expression in AML cells compared with that in normal cells (**Figure 1C**; **Supplementary Figures S2D, E**). The survival results showed that elevated expression of these genes (except *EXT2* and *PLD1*) was related to poorer prognosis (**Figure 1D**).

Next, the TCGA dataset was selected as the training set to examine the prognostic significance of these 13 genes in AML. Kaplan–Meier analysis indicated that high-risk patients had a poorer prognosis than the low-risk patients in the TCGA dataset (**Figure 1E**). Further, time-dependent ROC curves were used to validate the survival prediction model. The specificity and sensitivity of AUC in AML was > 0.7 even at six years and > 0.8 at 5 years (**Figure 1F**). Further, GSE37642-96, GSE71014, and GSE106291 databases were used as validation sets to validate the prognostic model. These results were consistent with the results of the training set. The AUCs at five years were a minimum of 0.5, showing an upward trend over time (**Figures 1G, H**). In addition, the PRC value analysis results were consistent with the ROC analysis results (**Supplementary Figure S3**).

### TRGs risk for clinical characteristics and prognosis in AML

**Figure 2:**
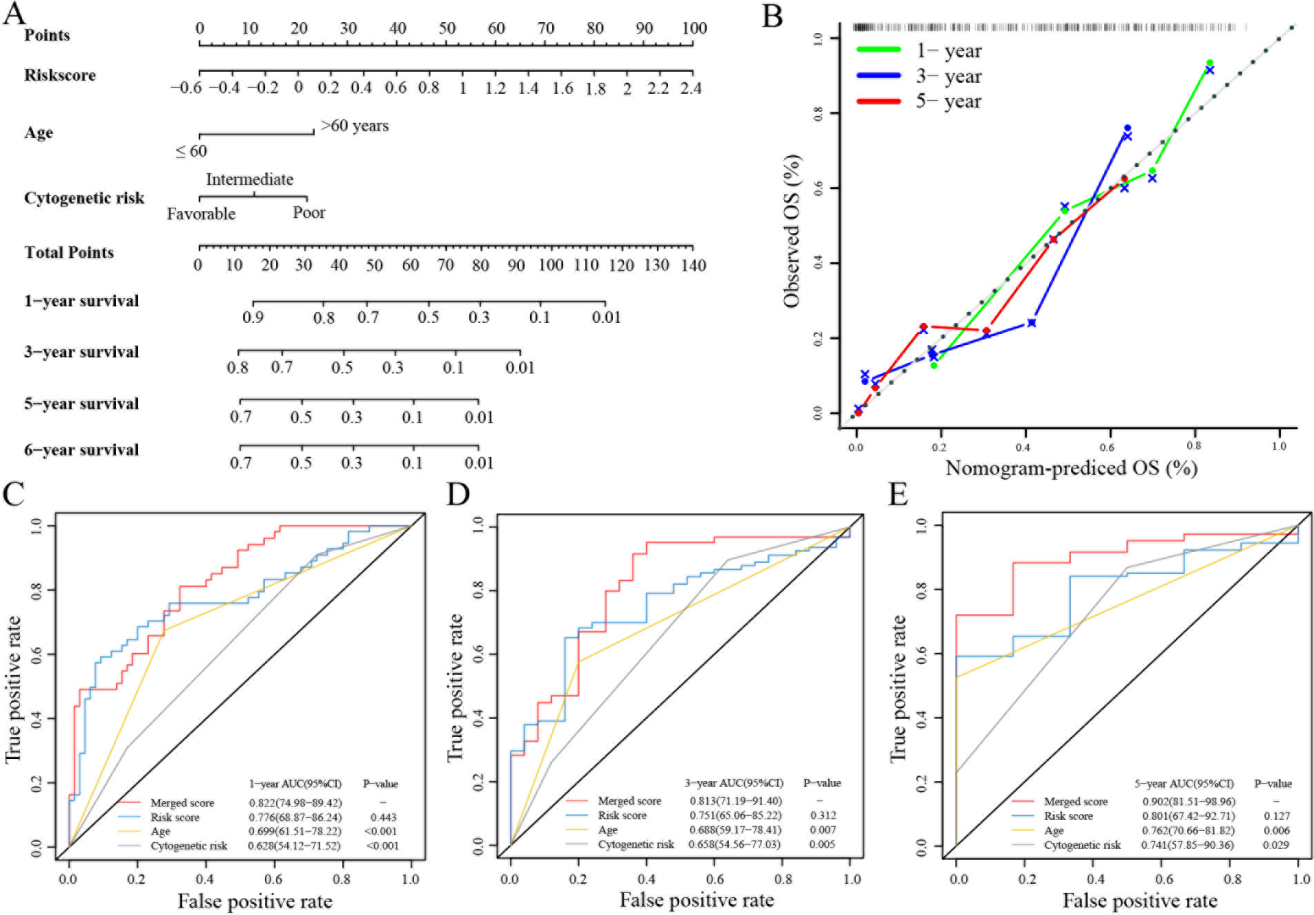
Building and validation of the nomogram to predict the OS of AML patients in the TCGA-LAML cohort. **(A)** Nomogram was built based on age, cytogenetic risk and risk score in the TCGA-LAML cohort. **(B)** Calibration plot of the nomogram. **(C-E)** Time-dependent receiver operating characteristic (ROC) curves of nomograms were compared based on 1-, 3- and 5-year OS of the TCGA-LAML cohort.

Next, a nomogram with standard clinical variables and the TRGs risk score was created to expand the clinical applicability in AML (**Figure 2A**). The score of each patient was calculated by combining each prognostic criterion. These results indicated that patients with higher total scores had poorer prognosis. The nomogram predicted that OS was highly consistent with the observed OS of the ideal model and predicted the 1-, 3- and 5-year survival time (**Figure 2B**). Specifically, the AUC for 1-year OS in the merged score group was 0.822 [95% CI: 74.98 – 89.42], in the risk score group was 0.776 [95% CI: 68.87 – 86.24], in age was 0.699 [95% CI: 61.51 – 78.22], and in cytogenetic risk was 0.628 [95% CI: 54.12 – 71.52]. Also, the AUC for 3-year survival in the merged score group was 0.813 [95% CI: 71.19 – 91.40], in the risk score group was 0.751 [95% CI: 65.06 – 85.22], in age was 0.688 [95% CI: 59.17 – 78.41] and in the cytogenetic risk was 0.658 [95% CI: 54.56 – 77.03]. Further, the AUC for 5 years OS in the merged score group was 0.902 [95% CI: 81.51 – 98.96], in the risk score group was 0.801 [95% CI: 67.42 – 92.71], in age was 0.762 [95% CI: 70.66 – 81.82], and in the cytogenetic risk was 0.741 [95% CI: 57.85 – 90.36]. These results demonstrated that subjoining the risk score significantly improved overall survival prediction efficiency (**Figures 2C-E**).

### Immune therapeutic targets of TRGs in AML

**Figure 3:**
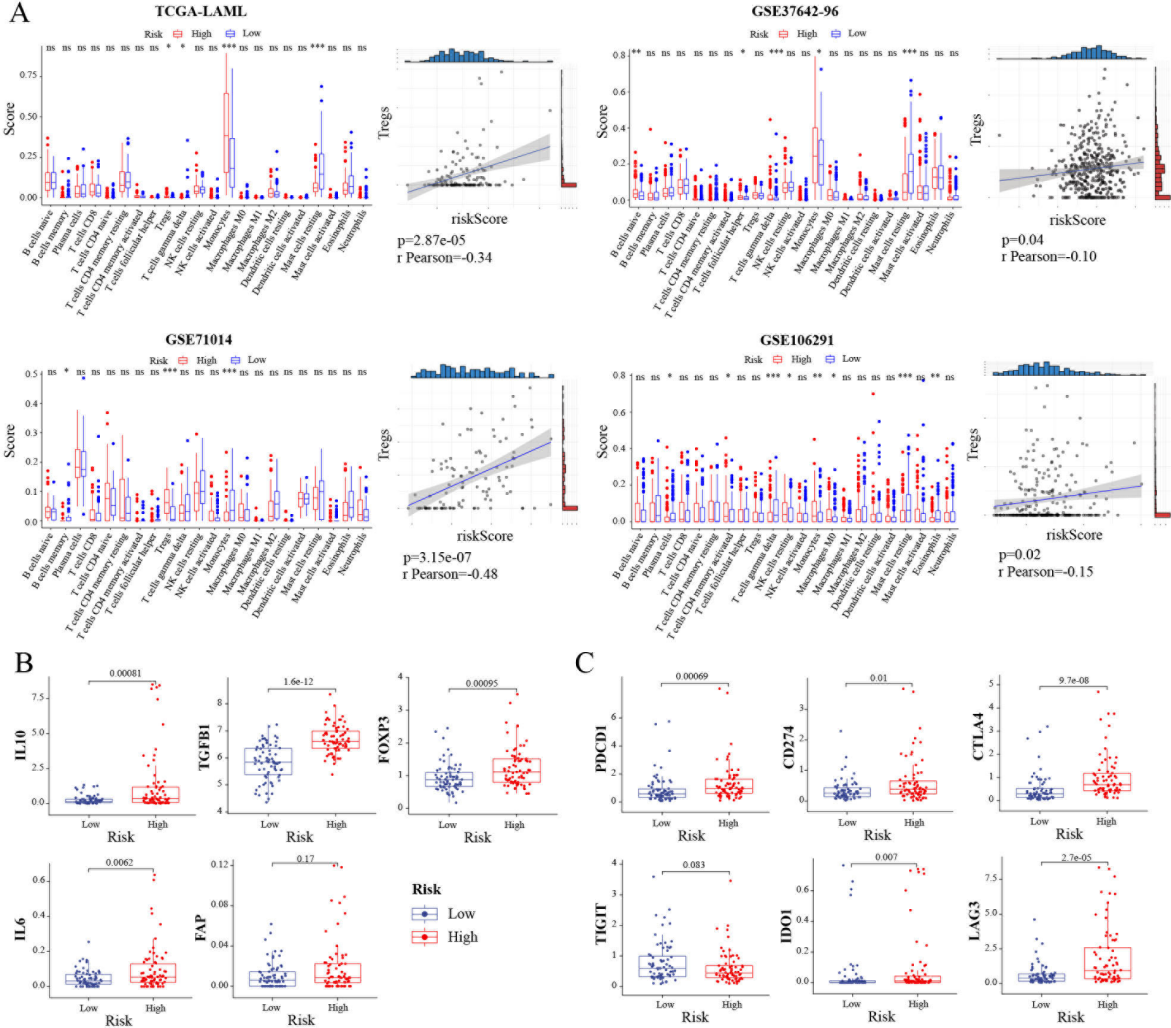
Landscape of tumor immune microenvironment between the high- and low-risk groups. **(A)** The scores of 22 immune cells and the correlation between riskscore and Tregs in the TCGA-LAML, GSE37642-96, GSE71014 and GSE106291 cohorts, respectively. **(B)** Investigations of tumor microenvironment immunosuppressive cytokines and markers. Expression level of IL10, TGFB1, FOXP3, IL6, and FAP in TCGA-LAML cohort. **(C)** Expression level of immune checkpoints in TCGA-LAML cohort. *, p < 0.05; **, p < 0.01; ***, p < 0.001.

Recent studies have substantiated the immense growth in tumor immunotherapy (12). Thus, the relationship between TRGs risk and infiltrated tumor microenvironment in AML was investigated. The results showed that there was a significant difference in the immune scores between the high-risk group and the low-risk group in the (i) Treg, T cells gamma delta, Monocytes, and Mast cells resting in the TCGA-LAML database; immune scores of B cells memory, Treg, and Monocytes in the GSE71014 database; immune scores of B cells naïve, T cells follicular helper, T cells gamma delta, Monocytes, and Mast cells resting in the GSE37642-96 database and immune scores of Plasma cells, T cells CD4 memory activated, T cells gamma delta, NK cells resting, Monocytes, Macrophages M0, Mast cells resting, and Eosinophils in the GSE106291 database. Monocytes were the common cells in all four databases. Also, Treg cells were closely associated with TRGs risk in these databases (**Figure 3A**). Next, the relation between the TRGs risk score and common immunosuppressive marker molecules in AML was investigated. The results showed that the expression of four immunosuppressive markers (IL-10, FOXP3, TGFB1, IL-6, except FAP) was upregulated in the high TRGs risk group compared with that in the low TRGs risk group (**Figure 3B**). Also, the correlation between the TRGs risk score and common immunotherapy targets, such as PDCD1, TIGHT, IDO1, CD274, CTLA4, and LAG3, was investigated. Consistent with our predictions, except TIGIT (p = 0.083), the other targets PDCD1 (p = 0.00069), IDO1 (p = 0.007), CD274 (p = 0.01), CTLA4 (p = 9.7e-08), and LAG3 (p = 2.7e-05) showed elevated expression in the high TRGs risk group (**Figure 3C**). Therefore, combining chemotherapy and current immunotherapy-related drugs could improve the prognosis of patients in the high TRGs risk group.

### Intestinal flora 16S sequencing

The change in the intestinal flora can affect the prognosis of patients with leukemia (18–20). Tigecycline, a broad-spectrum antibiotic, has the potential to induce intestinal flora disorders. Thus, 16S rRNA sequencing was done to investigate the microbial community changes regulated by tigecycline. The classified composition of microorganisms and different classification units were used in the two groups before and after applying tigecycline (**Supplementary Figure S4**). Ggtree was used to display the position of ASV/OTU in the evolutionary tree and the mutual evolution distance. Their composition and abundance, classification, and other information were visualized via hot charts and column diagrams (**Supplementary Figure S5**). The results of the alpha diversity index suggested that there was no apparent difference between the two groups (**Supplementary Figure S6**).

**Figure 4:**
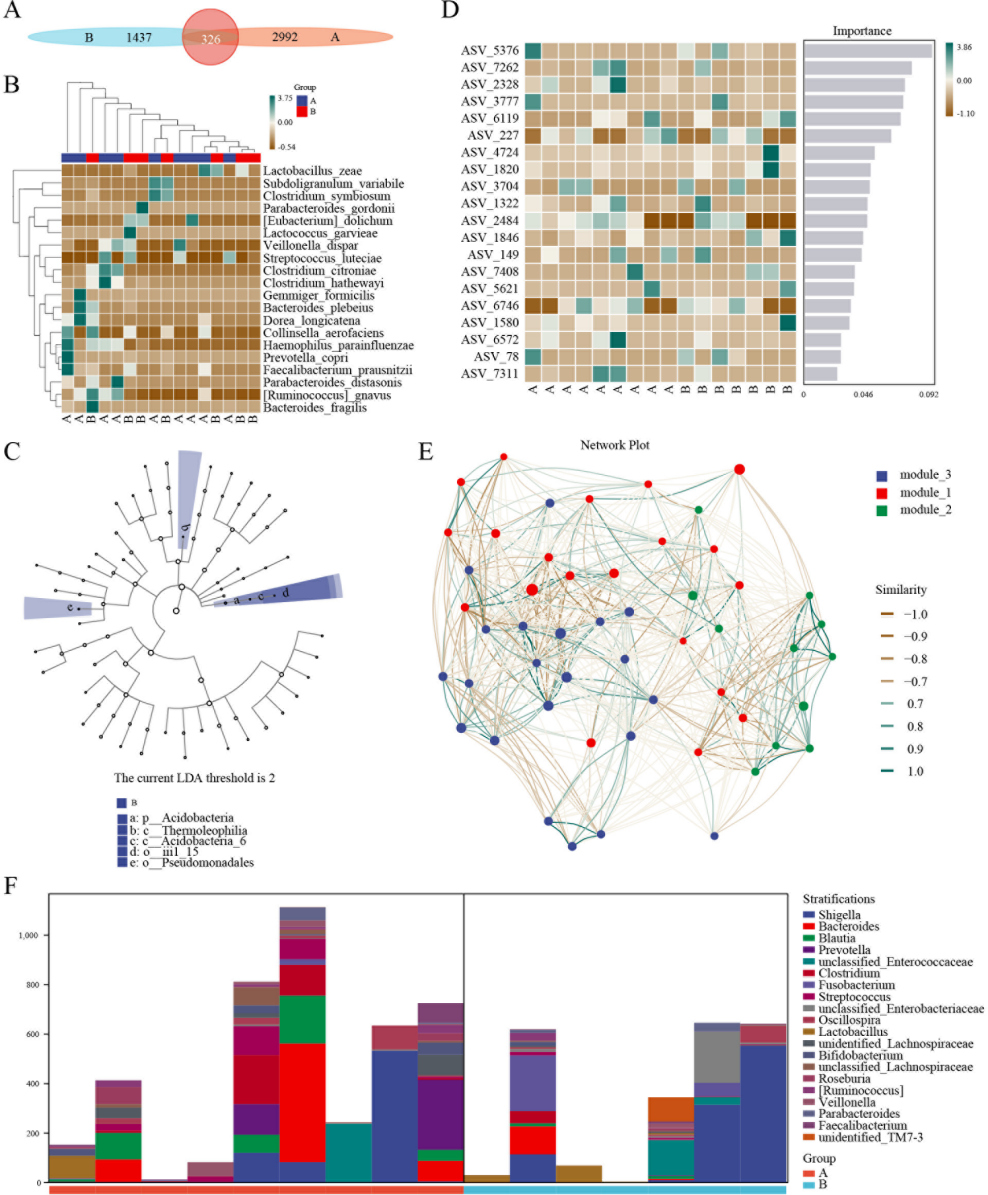
16S sequencing of AML patients treated with tigecycline. **(A)** ASV/OTU abundance analysis of patients before or after tigecycline treatment. **(B)** Hot diagrams of species composition in the trend of species. **(C)** LEFSE analysis showed the consistency of the microbiology group. **(D)** Hot diagrams of complex non-linearly dependencies between samples and groups. **(E)** The time and space microorganized community analysis between microorganisms. **(F)** The metabolic pathway analysis of stratification between the two groups.

In contrast, the beta diversity analysis indicated the presence of significant differences between the two groups of microbial communities (**Supplementary Figure S7**). Next, ASV/OTU abundance tables were used to make petal diagrams for community analysis to further explore the differences between the species and to study which species were common and which were unique. After tigecycline treatment, a significant change was observed in the community composition. Only 326 species in the two groups were common (**Figure 4A**). Also, a significant difference in the trend of species composition of hot diagrams was observed (**Figure 4B**). Further, LEFSE was used to explore the search for stable different species between the two groups and to identify the consistency of the microbiology group that showed similar performance in the two groups. There were significant differences in the presence of p_Acidobacteria, c_Thermoleophilia, c_Acidobacteria_6, o_iii1_15, and o_Pseudomonadales (**Figure 4C**; **Supplementary Table S2**). A classic and efficient machine learning algorithm (Random forest analysis) deepened the complex non-linear dependencies between samples and groups. The critical indicators of the top 5 were ASV_5376, ASV_7262, ASV_2328, ASV_3777, and ASV_6119, respectively (**Figure 4D**). Next, a specific microorganized community was identified in time and space via associated analysis, and a related network was constructed to determine the relationship between microorganisms. The network showed that these three modules were intertwined and closely connected. Also, the Zi and PI values showed that the network nodes (ASV/OTU) were divided into 4 parts. Most phylum (Firmicutes, Proteobacteria, Bacterodetes, and Actinobacteria) landed on the part of the Peripherals (**Figure 4E**).

For microbial ecology research, the functional potential of the flora was investigated. The analysis of metabolic pathways suggested that the primary functions included biosynthesis of amino acid and nucleoside and nucleotide, degradation of carbohydrate and carboxylate, fermentation, and glycolysis (**Supplementary Figure S8**). The layered sample metabolic pathway abundance tables were also used to analyze the pathway species. The results showed significant differences in stratification between the two groups and even in different samples (**Figure 4F**).

## Discussion

Tigecycline has a broad antibacterial spectrum with intense activity for Gram-positive and Gram-negative bacteria (21, 22). It is widely used to treat complex infections in patients with AML (23). The most common adverse reactions are discomfort in the digestive system, including fatigue, nausea, vomiting, diarrhea, and abdominal pain. The symptoms are mostly mild to moderate and disappear after the drug is discontinued (24). With the increase in clinical applications of tigecycline, there are increasing reports of abnormal coagulation function in non-tumor patients (25). This group has previously reported that using tigecycline to control complicated infections in tumor patients can cause apparent coagulation function abnormalities, especially in patients with AML (13, 26). However, the mechanism causing coagulation dysfunction was not clear.

Tigecycline is believed to inhibit vitamin K synthesis, affect coagulation factor synthesis, or inhibit the IL-6 mechanism to induce coagulation disorders (26, 27). Early studies in CHO cells revealed that tigecycline affected the changes in the internal genetic expression of the cells and affected the adhesion function of platelets. According to the genetic construction risk model based on the genes regulated by tigecycline and through analysis and verification of multiple databases, this study revealed that the prognosis of patients with high-risk group characteristics was significantly poorer.

The role of platelet function-related genes in tumor prognosis has drawn significant attention (28). Patients with triple-negative breast cancer TNBC could be divided into three subtypes according to platelet-related gene expression and variation levels (29). Many platelet-related genes in pancreatic ductal adenocarcinoma (PDAC) were significantly enriched in CTCs (30). The expression of HLA-E molecules on the tumor cell surface was upregulated to escape the immune surveillance of NK cells. Moreover, prognostic models of ovarian cancer patients have been established using tumor-domesticated platelets (TEP), which are used for early cancer screening and prognostic prediction (31, 32). Earlier, the authors of this study reported for the first time that tigecycline affected the procoagulant function by regulating the changes of hematopoietic related genes. In this manuscript, the authors report the first diagnosis model of platelet-related genes based on the regulation of tigecycline. Further, the prognostic prediction efficiency of this model was superior to the existing traditional methods in AML patients. Moreover, the combination of this model with conventional models can further improve the accuracy of AML prediction.

The immune microenvironment and microecological changes in the intestine are closely related to the tumor (33). There are several bacteria in the intestinal tract, and maintaining this homeostasis requires the participation of Treg on the one hand and immune cells to resist bacterial invasion on the other hand. Intestinal flora was reported to regulate the Th17/Treg cell differentiation in a mouse model of experimental autoimmune prostatitis by short-chain fatty acid propionic acid in various tumor types (34). TLR4 plays a sexual role in mice colitis by promoting the colonization of Akk bacteria in the intestine and upregulating the RORγt+ Treg cell-mediated immunosuppressive response (35). RORγt+ cells induce intestinal flora-specific differentiation of Treg cells (36). In this study, the application of tigecycline in patients with AML significantly affected the proportion and function of Treg cells in the body, which in turn caused changes in the intestinal flora and the prognosis of patients.

**Figure 5:**
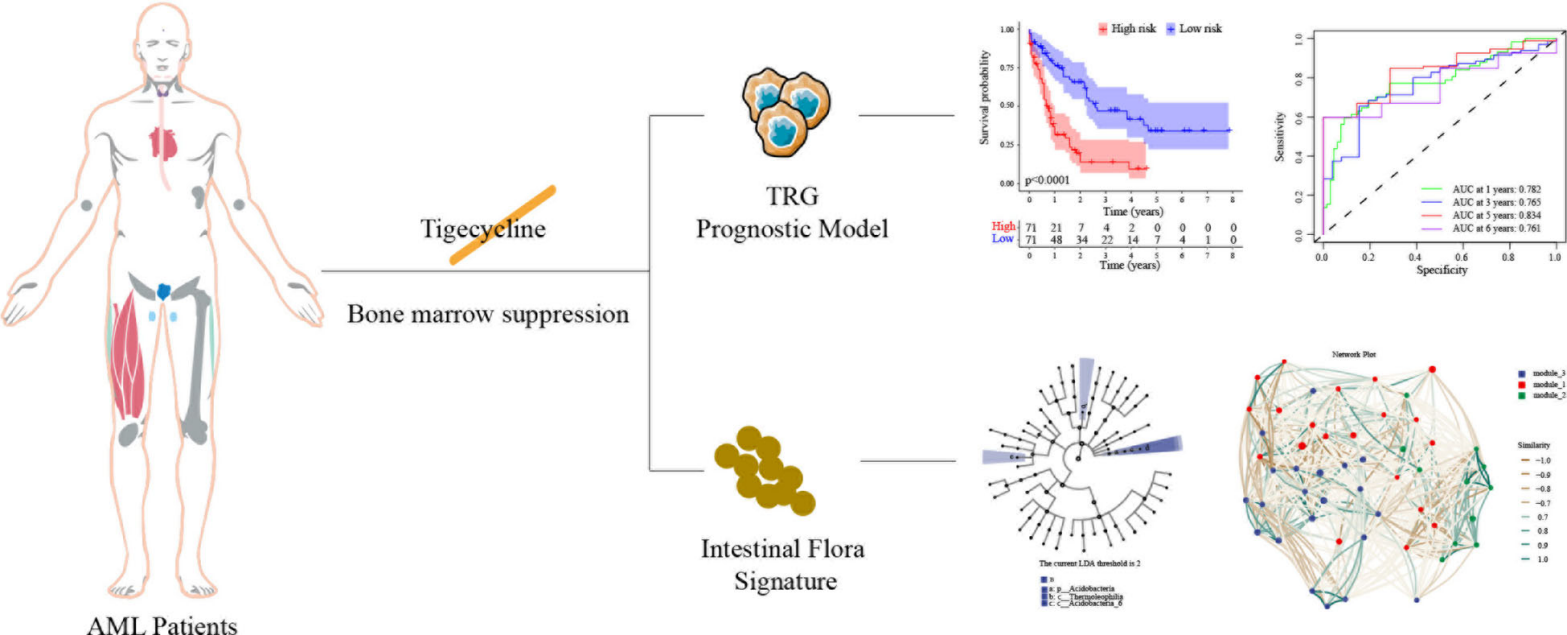
A conclusive hypothesis of the influence of tigecycline in AML patients.

While there is no direct evidence at present to establish a connection between these microbial shifts and patient prognosis, a growing number of studies indicate that changes in the gut microbiome of individuals with AML are a contributing factor to their prognostic outcomes. AML patients receive multiple antibiotic treatments during induction chemotherapy, causing significant damage to the gut microbiota (37). In fact, our results indicated that tigecycline could also kill too many bacterial species in the intestinal microbiota, leading to alterations in bacterial diversity. For long-term treatment, reduced “good” microbiota could be troublesome such as increased “bad bacteria” infection potential and noncoding RNA dysfunctions linked to mitochondrial dysfunctions and AML. In light of this, we recommend exercising caution rather than imposing a ban on the use of tigecycline in patients experiencing bone marrow suppression. Moreover, we are committed to pursuing additional research to elucidate the intricate relationship between these elements. After chemotherapy and multiple antibiotic treatments, if the gut microbiota of AML patients wants to return to the initial antibiotic treatment state, it is necessary to reduce antibiotic pressure earlier or undergo fecal microbiota transplantation (**Figure 5**). Due to the stringent regulatory constraints on advanced antimicrobials such as tigecycline, rapid acquisition of additional samples is not feasible. Consequently, we are contemplating experimental protocols to assess the implications of our research findings across various neoplastic entities and patient prognoses. Our objective is to conduct a controlled clinical trial to substantiate the hypotheses advanced in further study.

## Data Availability

The datasets presented in this study can be found in online repositories. The names of the repository/repositories and accession number(s) can be found in the article/**Supplementary Material**.

## Supplementary material

Supplementary Figure 1 A flowchart of data processing.

Supplementary Figure 2
**(A)** The volcano plot analysis of DEGs in GSE1159 cohort. **(B)** Heatmap of 34 DEGs in GSE1159 cohort. **(C)** The circos plot depicting the location on chromosomes of 13 prognostic DEGs. **(D)** The CNV frequency of 13 prognostic DEGs in TCGA-LAML cohort. **(E)** Genetic alterations of the 13 prognostic DEGs in CCLE, obtained from the cBioportal for Cancer Genomics (http://www.cbioportal.org/).

Supplementary Figure 3 PRC curve analysis of AML databases.

Supplementary Figure 4 The classification composition of microorganisms and different classification units in the groups before and after the application of tigecycline.

Supplementary Figure 5 Phylogenetic tree plot analysis of microorganisms in the groups before and after the application of tigecycline.

Supplementary Figure 6 Alpha diversity index analysis of microorganisms in the groups before and after the application of tigecycline.

Supplementary Figure 7 Hierachical clustering analysis of microorganisms in the groups before and after the application of tigecycline.

Supplementary Figure 8 Function analysis of microorganisms in the groups before and after the application of tigecycline.

Supplementary Figure 9 Validation of CRGs genes expression in AML cell line THP cells treated with tigecycline.

Supplementary Table 1 The primers of CRGs by qPCR.

Supplementary Table 2 The list of LEFSE analysis in the microbiology group.
