# Supplementary figures and images for: Tigecycline-induced coagulation gene prognostic prediction model and intestinal flora signature in AML
Source: Front Immunol. 2024 Nov 14;15:1486592. doi: 10.3389/fimmu.2024.1486592 (PMC11602473; doi:10.3389/fimmu.2024.1486592)

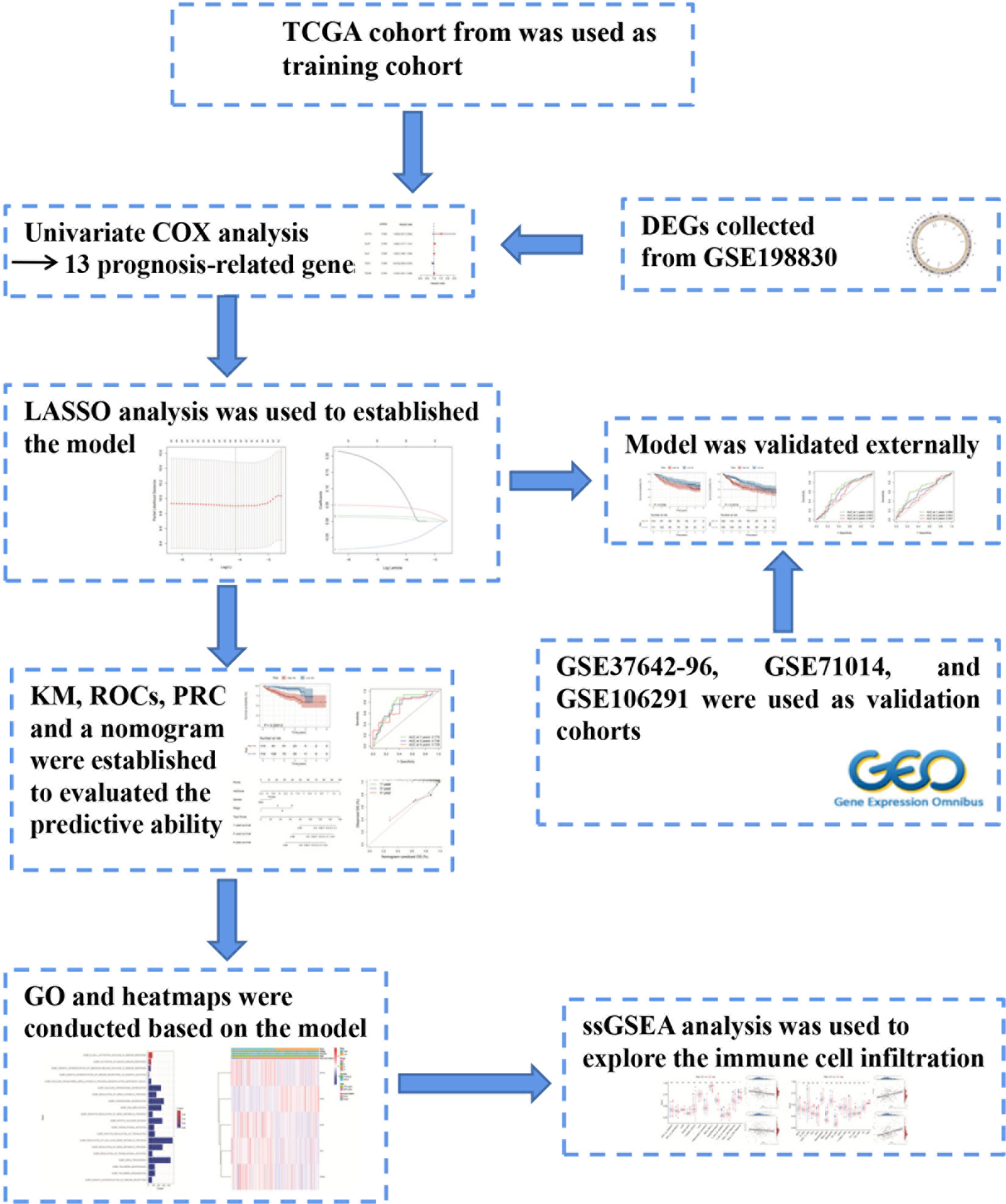

Supplement: Supplementary Figure 1 — A flowchart of data processing. [file Image1.tif]

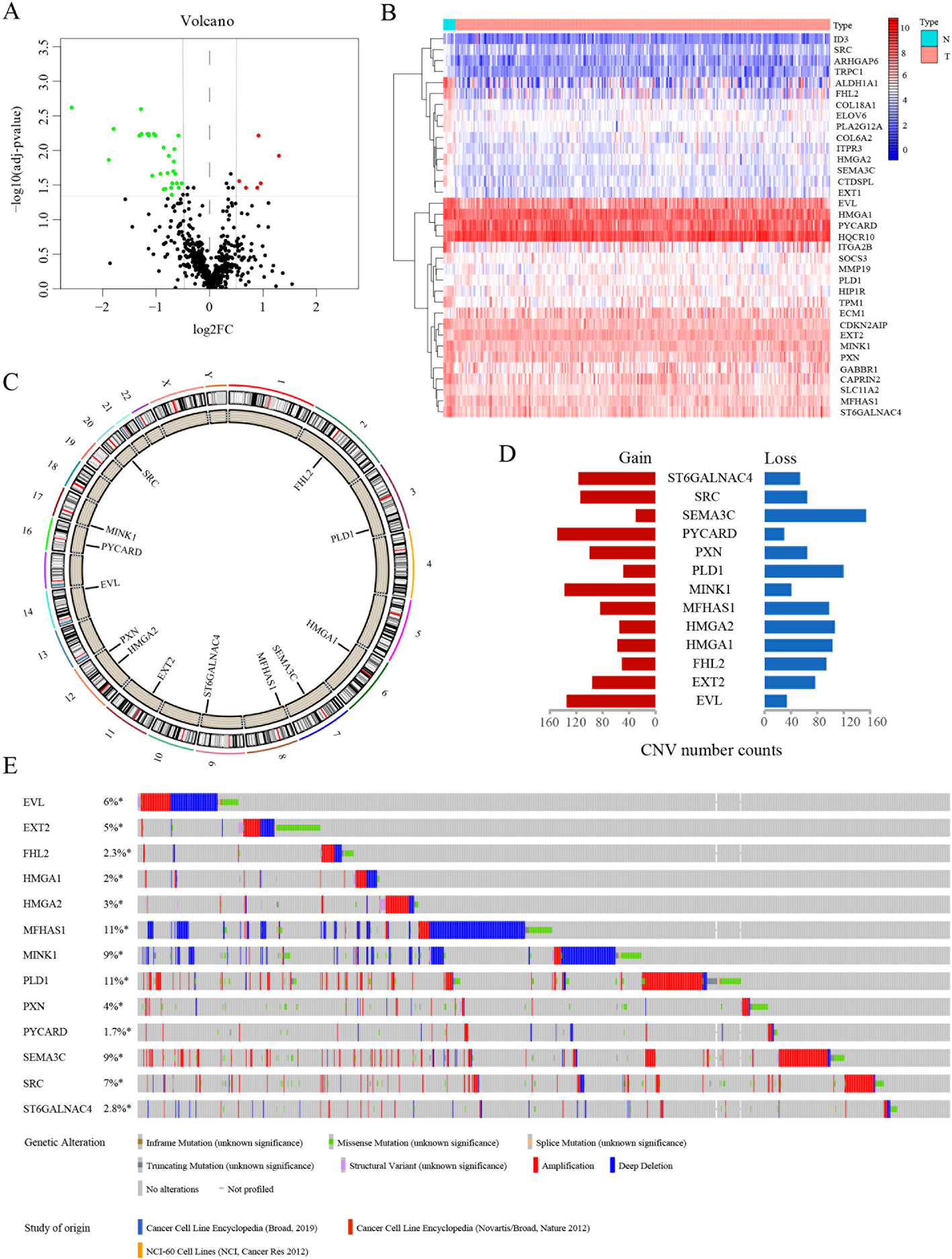

Supplement: Supplementary Figure 2 — (A) The volcano plot analysis of DEGs in GSE1159 cohort. (B) Heatmap of 34 DEGs in GSE1159 cohort. (C) The circos plot depicting the location on chromosomes of 13 prognostic DEGs. (D) The CNV frequency of 13 prognostic DEGs in TCGA-LAML cohort. (E) Genetic alterations of the 13 prognostic DEGs in CCLE, obtained from the cBioportal for Cancer Genomics (http://www.cbioportal.org/). [file Image2.tif]

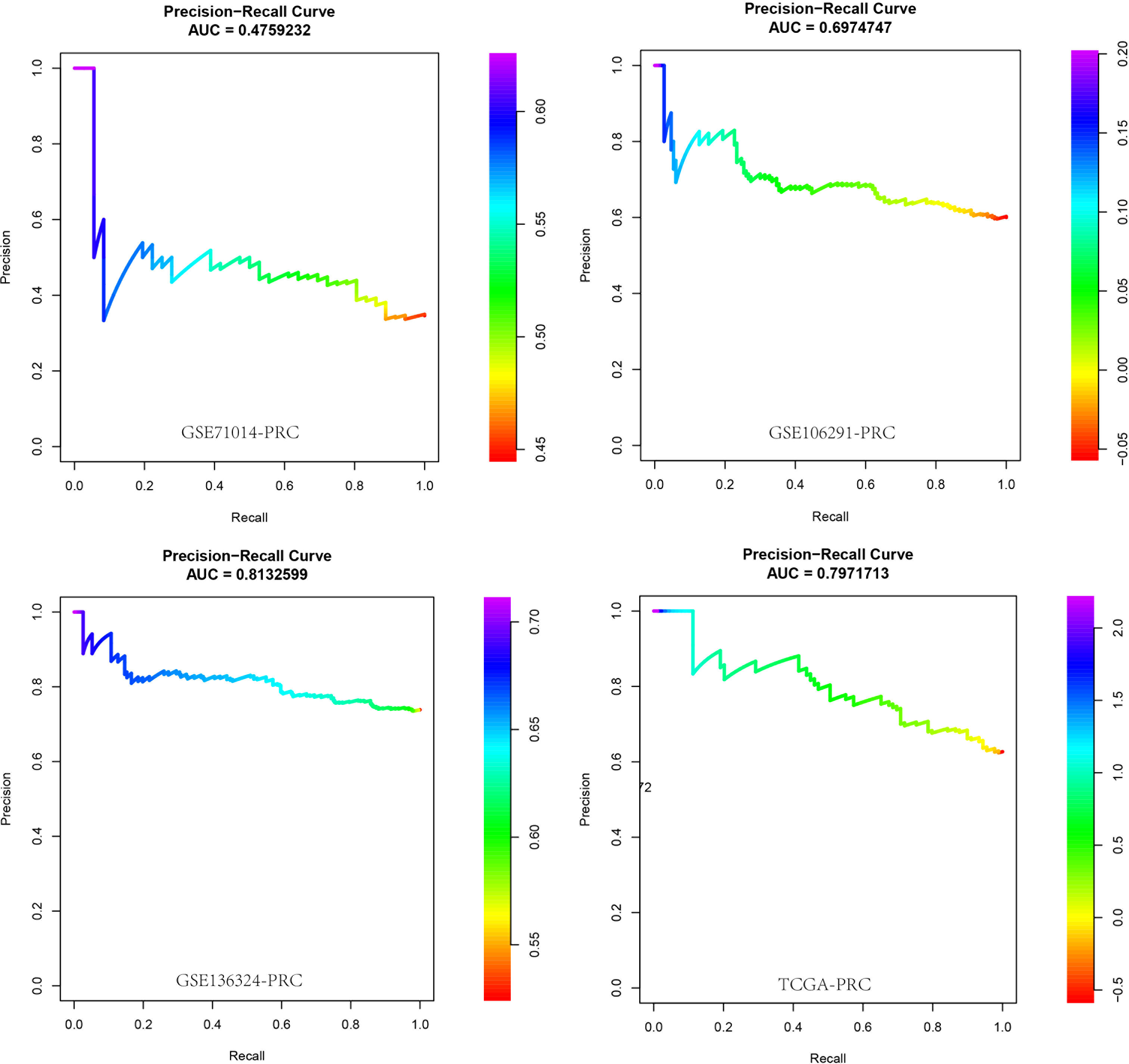

Supplement: Supplementary Figure 3 — PRC curve analysis of AML databases. [file Image3.tif]

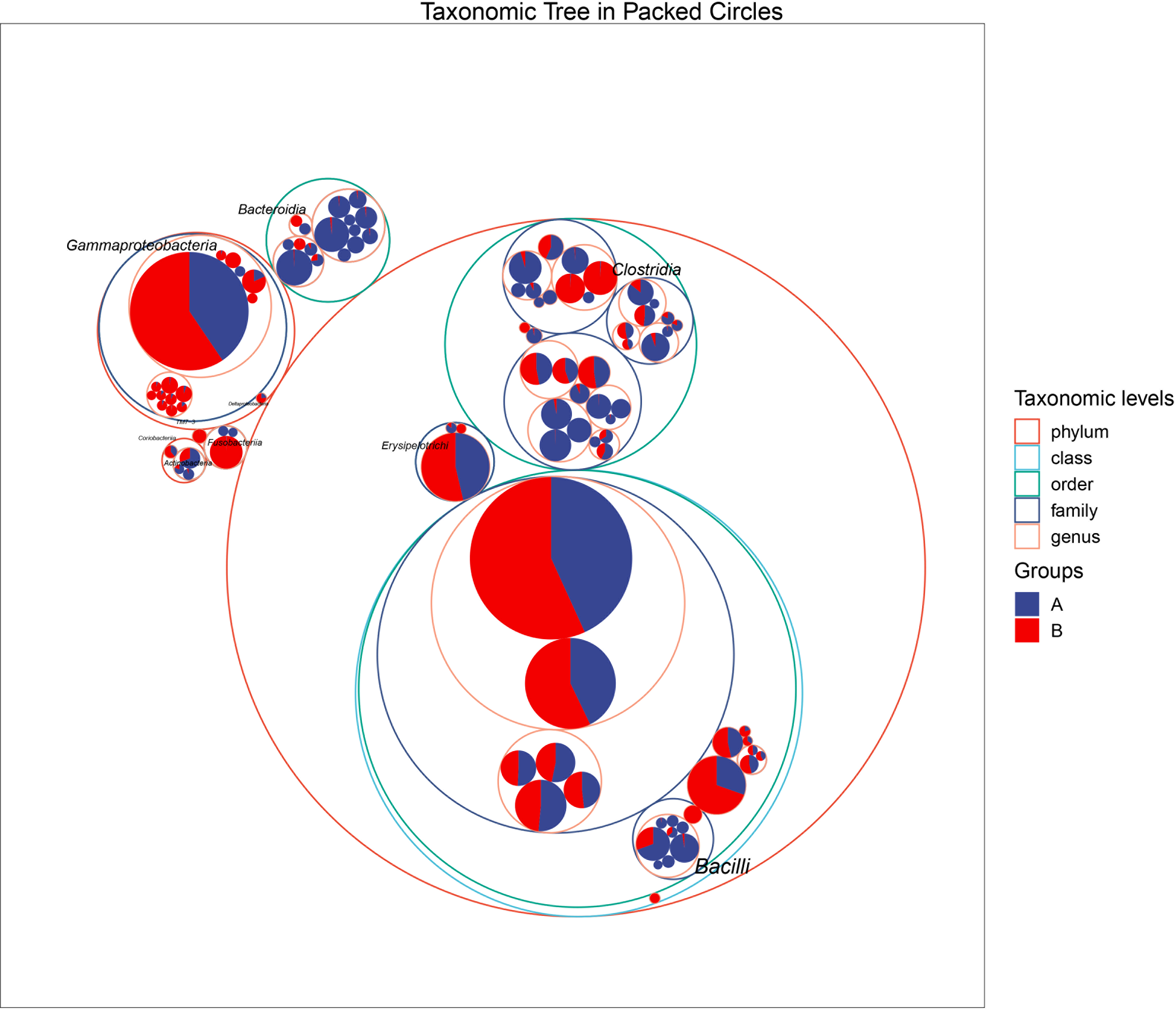

Supplement: Supplementary Figure 4 — The classification composition of microorganisms and different classification units in the groups before and after the application of tigecycline. [file Image4.tif]

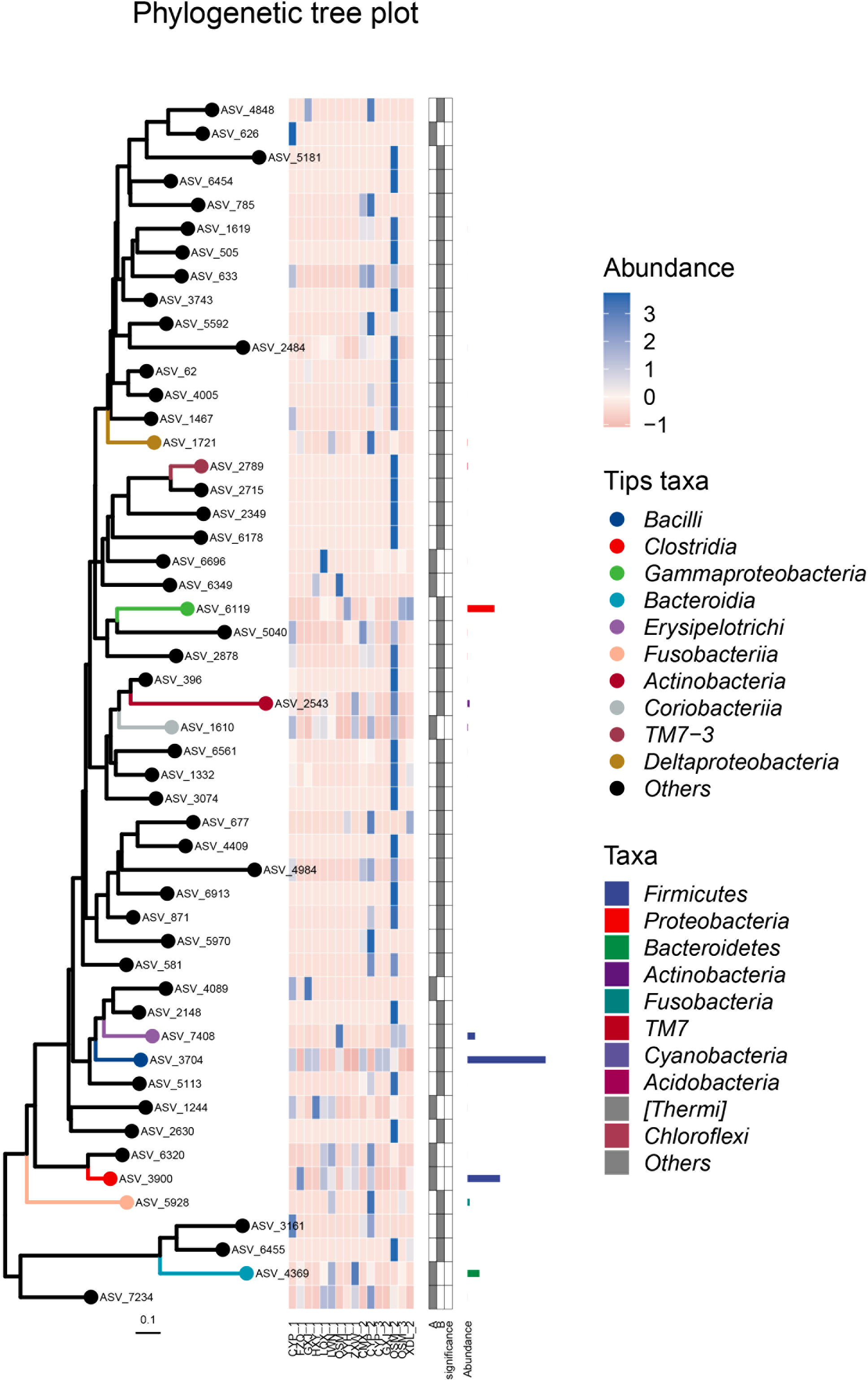

Supplement: Supplementary Figure 5 — Phylogenetic tree plot analysis of microorganisms in the groups before and after the application of tigecycline. [file Image5.tif]

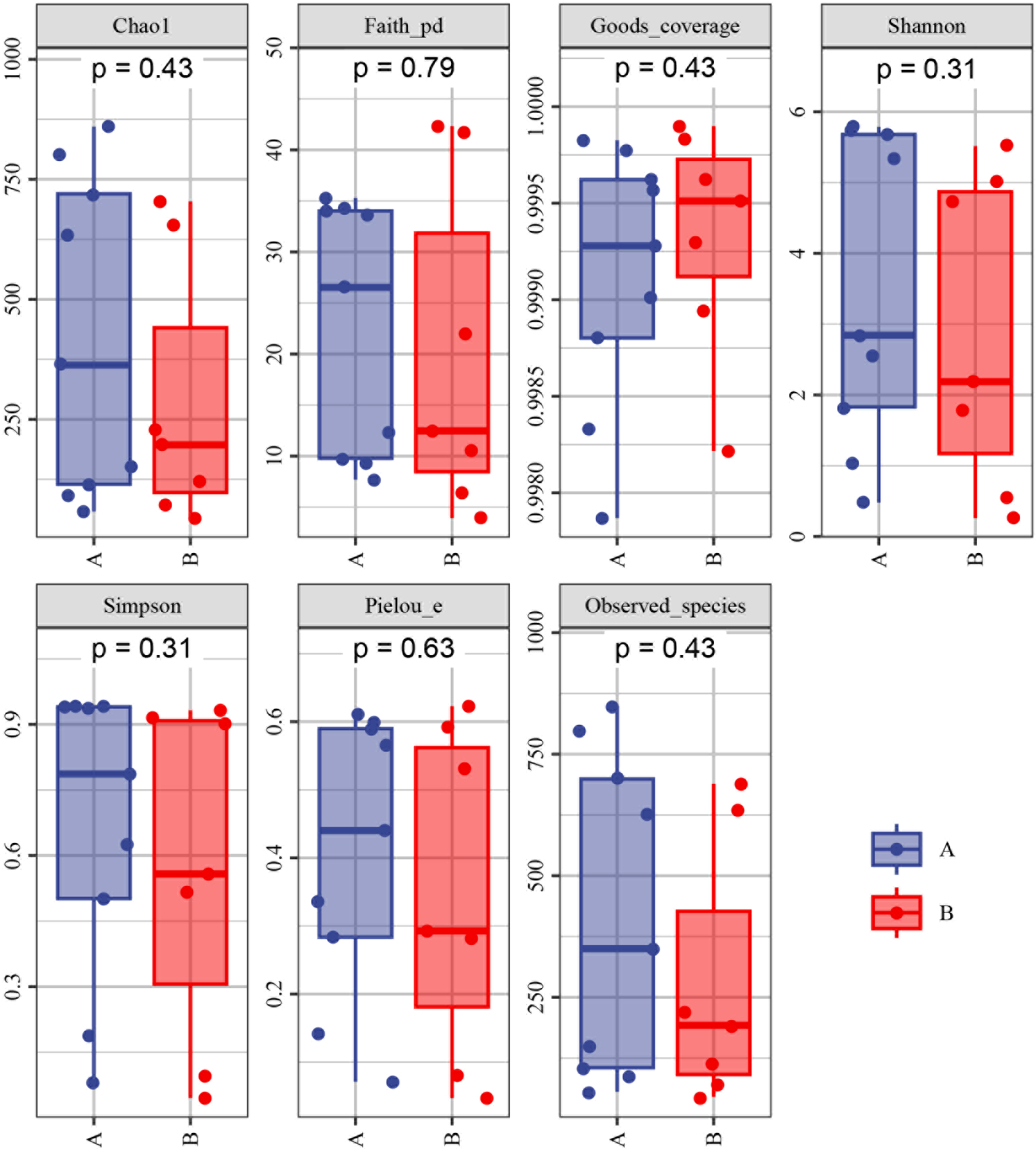

Supplement: Supplementary Figure 6 — Alpha diversity index analysis of microorganisms in the groups before and after the application of tigecycline. [file Image6.tif]

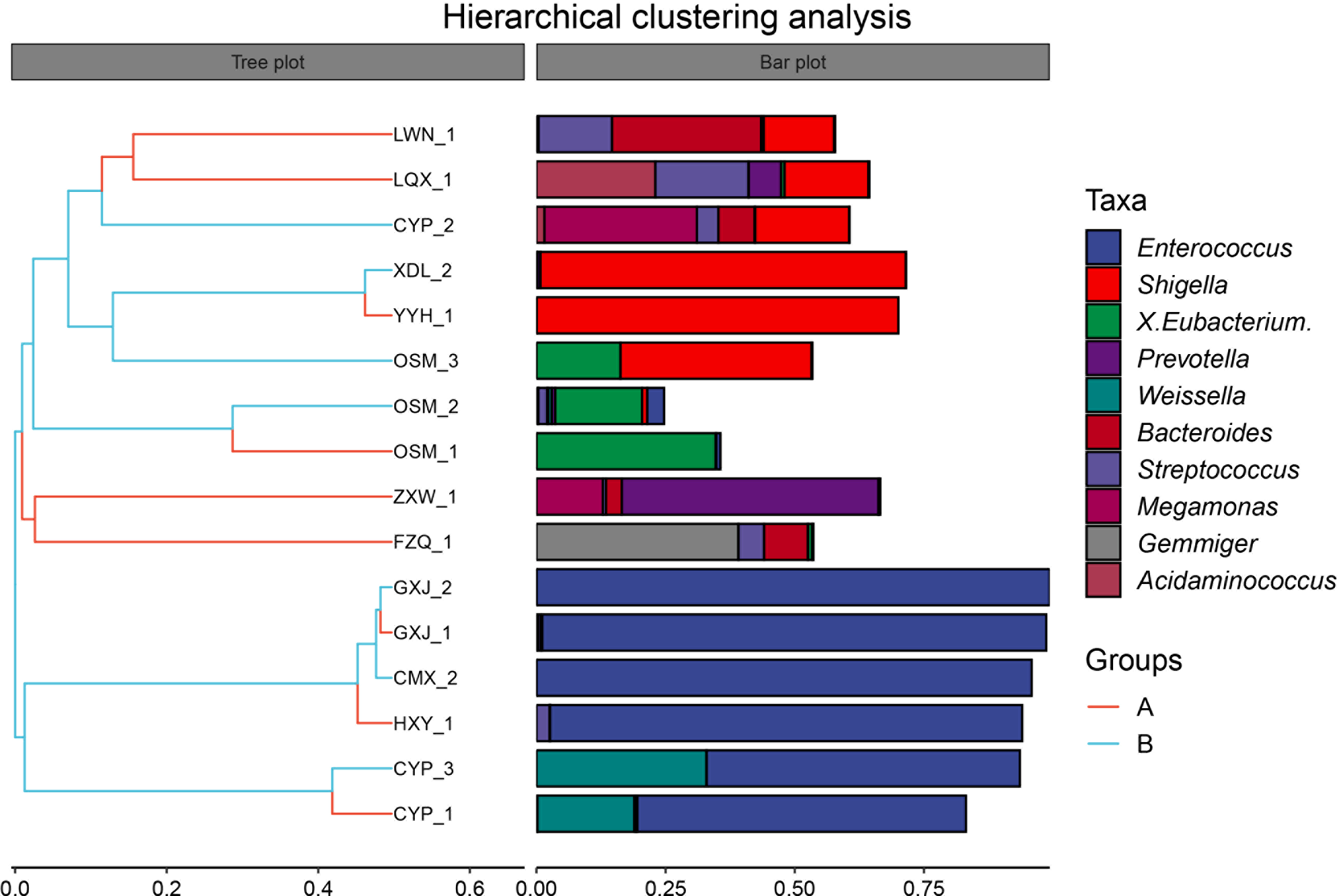

Supplement: Supplementary Figure 7 — Hierachical clustering analysis of microorganisms in the groups before and after the application of tigecycline. [file Image7.tif]

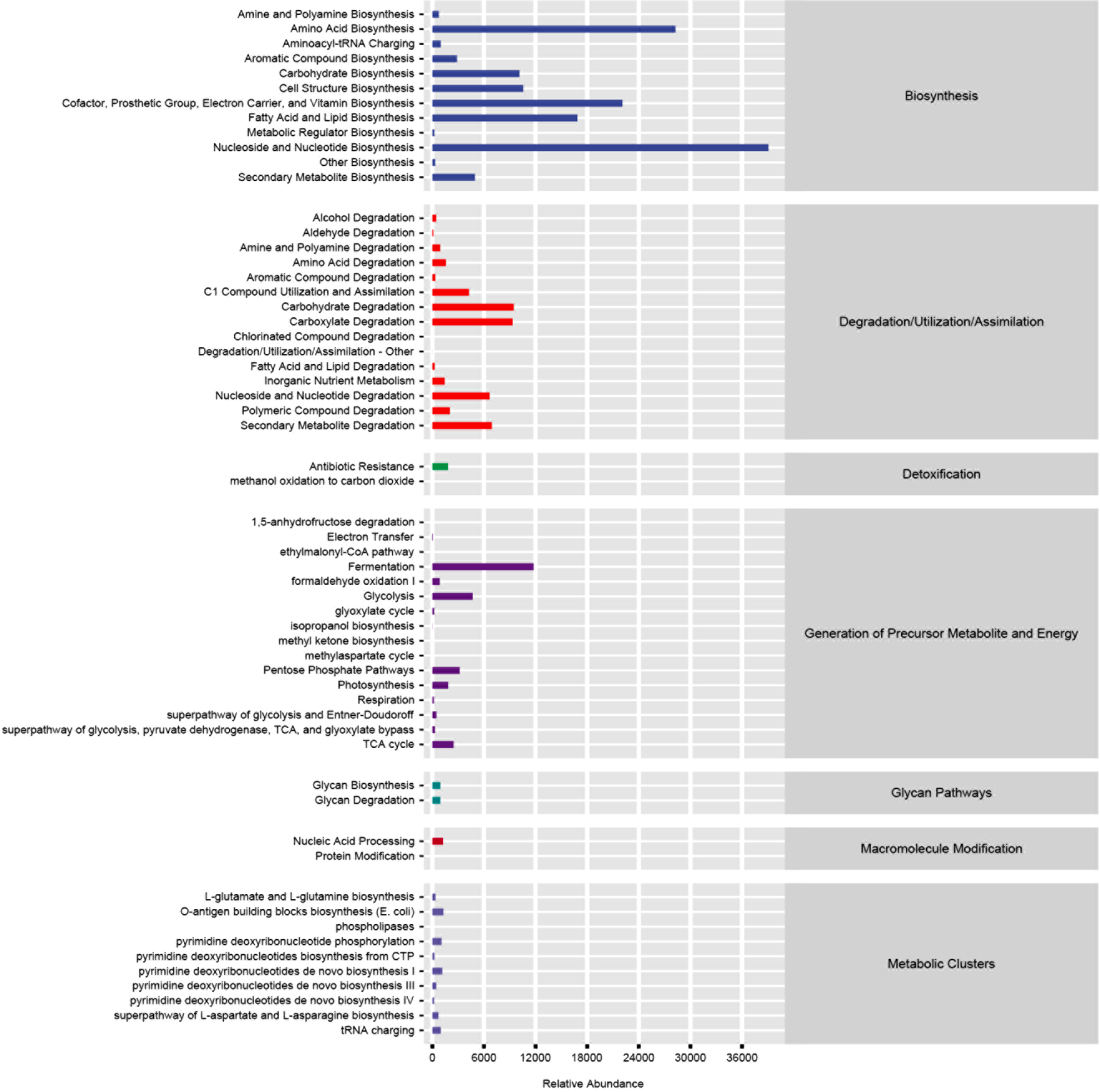

Supplement: Supplementary Figure 8 — Function analysis of microorganisms in the groups before and after the application of tigecycline. [file Image8.tif]

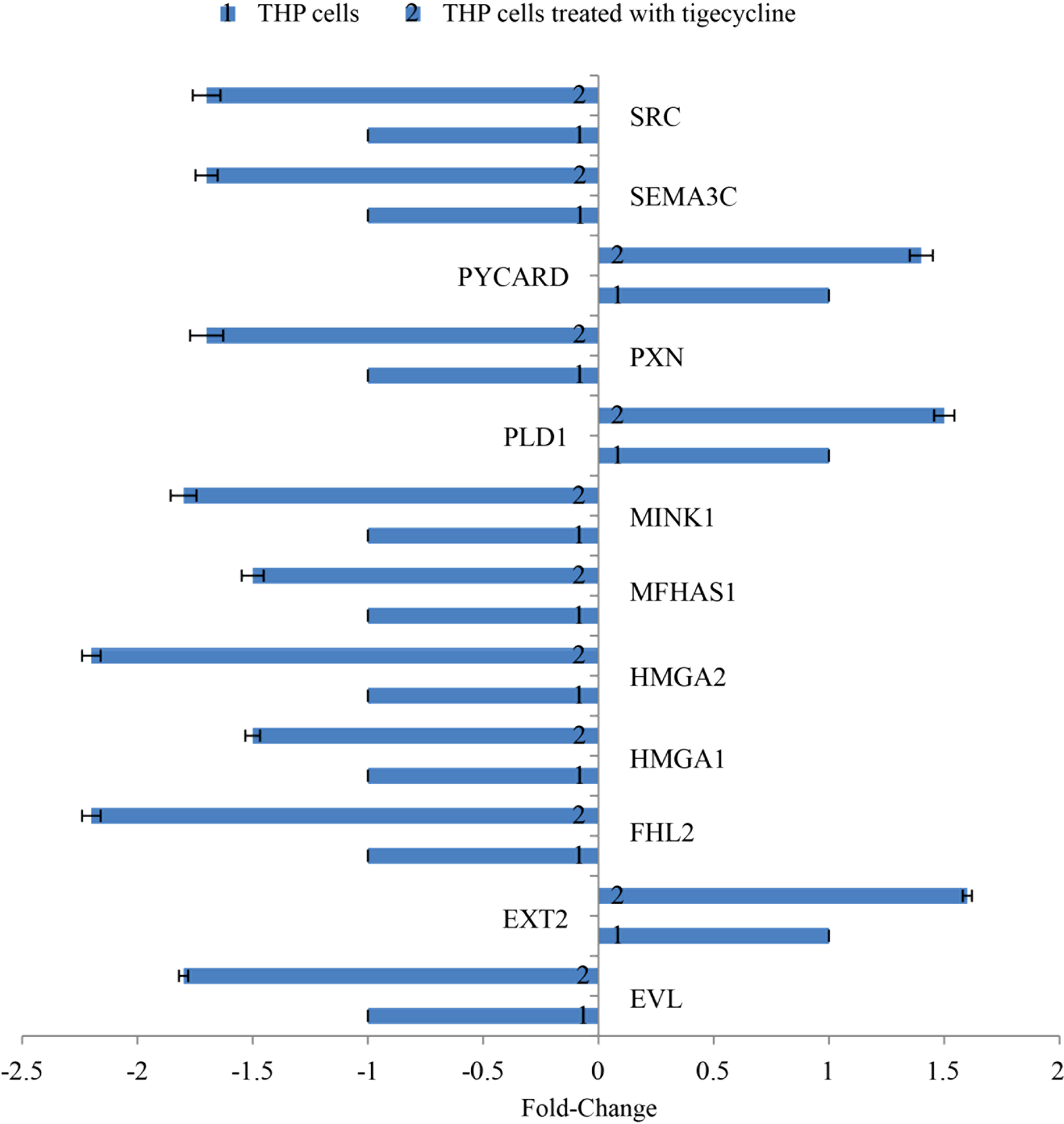

Supplement: Supplementary Figure 9 — Validation of CRGs genes expression in AML cell line THP cells treated with tigecycline. [file Image9.tif]
