# Supplementary material for: Tigecycline-induced coagulation gene prognostic prediction model and intestinal flora signature in AML
Source: Front Immunol. 2024 Nov 14;15:1486592. doi: 10.3389/fimmu.2024.1486592 (PMC11602473; doi:10.3389/fimmu.2024.1486592)
Supplement: Supplementary file 12 [file DataSheet1.pdf]

```
#install.packages("survivalROC")
#if(!require("ggsci")) BiocManager::install("ggsci",updata=F,ask = F)
#if(!require("timeROC")) BiocManager::install("timeROC",updata=F,ask = F)


bioROC=function(riskFile=null,cliFile=null,outFile=null,year){
	risk=read.table(riskFile,header=T,sep="\t",check.names=F,row.names=1)
	cli=read.table(cliFile,sep="\t",check.names=F,header=T,row.names=1)
	sameSample=intersect(row.names(cli),row.names(risk))
	risk=risk[sameSample,]
	cli=cli[sameSample,]
	rt=cbind(futime=risk[,1],fustat=risk[,2],cli,riskScore=risk[,(ncol(risk)-1)])


	rt=rt[,c(grep("futime",colnames(rt)),
		grep("fustat",colnames(rt)),
		grep("riskScore",colnames(rt)),
		grep("IPI",colnames(rt)))]
	rt$id=rownames(rt)


	cox<- coxph(Surv(futime,fustat) ~ IPI,data =rt)
	score=predict(cox,type="risk",newdata=rt)
	score=cbind(id=rt$id,IPI=as.numeric(score))
	rt=rt[,-grep("IPI",colnames(rt))]
	rt=merge(rt,score,by="id")
	rt[,ncol(rt)]=as.numeric(rt[,ncol(rt)])

	cox<- coxph(Surv(futime,fustat) ~riskScore+IPI,data =rt)
	score=predict(cox,type="risk",newdata=rt)
	score=cbind(id=rt$id,Mergeed_score=as.numeric(score))
	rt=merge(rt,score,by="id")
	rt[,ncol(rt)]=as.numeric(rt[,ncol(rt)])

	rownames(rt)=rt$id
	rt=rt[,-grep("id",colnames(rt))]

	rocCol=jcopal
	colnames(rt)[grep("riskScore",colnames(rt))]="Risk score"

	for (k in 1:length(year)) {
		aucText=c()
		pdf(file=outFile[k],width=6.5,height=6.5)
		par(oma=c(0.5,1,0,1),font.lab=1.5,font.axis=1.5)
```

```
        ROC<- timeROC(T=rt$futime,
                      delta=rt$fustat,
                      marker=rt$Mergeed_score,
                      cause=1,
                      weighting="marginal",
                      times=c(1,3,5),
                      iid=TRUE)
        plot(ROC$FP[,k], ROC$TP[,k], type="l", xlim=c(0,1), ylim=c(0,1),col="#ED0000B2",
             xlab="False positive rate", ylab="True positive rate",
             lwd = 5,lty=1, cex.main=1.3, cex.lab=1.2, cex.axis=1.2, font=1.2)
        abline(0,1)
        confint=confint(ROC, level = 0.95)$CI_AUC
        key=paste(as.numeric(round(ROC$AUC[k],3)),"(",confint[k,1],"-",confint[k,2],")",sep="")
        aucText=c(aucText,paste("Merged score","                ",key,"            -",sep=""))

        j=0
        for(i in colnames(rt[,3:(ncol(rt)-1)])){
            ROC_cli<- timeROC(T=rt$futime,
                              delta=rt$fustat,
                              marker=rt[,i],
                              cause=1,
                              weighting="marginal",
                              times=c(1,3,5),
                              iid=TRUE)
            j=j+1
            lines(ROC_cli$FP[,k],        ROC_cli$TP[,k],        type="l",        xlim=c(0,1),
ylim=c(0,1),col=rocCol[j],
                  xlab="False positive rate", ylab="True positive rate",
                  lwd = 5,lty=1, cex.main=1.3, cex.lab=1.2, cex.axis=1.2, font=1.2)
            confint=confint(ROC_cli, level = 0.95)$CI_AUC

key=paste(as.numeric(round(ROC_cli$AUC[k],3)),"(",confint[k,1],"-",confint[k,2],")",sep="")
            P=compare(ROC_cli,ROC, adjusted = TRUE)

pvalue=ifelse(P$p_values_AUC[2,k]>=0.001,round(P$p_values_AUC[2,k],3),"<0.001")
            aucText=c(aucText,paste(i,"                ",key,"      ",pvalue,sep=""))
        }

        legend("bottomright",  c(paste("                              ",paste0(year[k],"-year
AUC(95%CI)"),"       ","P-value",sep=""),
                              aucText),
               col=c("white","#ED0000B2",rocCol),bty='n', lty=1, lwd=4.5, cex=0.95)
        dev.off()
    }
```

```
}
bioROC(riskFile="tcgaRisk.txt",cliFile="result-10846.txt",outFile=c("tcgaROC1(nomogram).pdf","tcgaROC3(nomogram).pdf","tcgaROC5(nomogram).pdf"),year=c(1,3,5))
bioROC(riskFile="geoRisk-4732.txt",cliFile="result-4732.txt",outFile=c("geoROC1(nomogram).pdf","geoROC3(nomogram).pdf","geoROC5(nomogram).pdf"),year=c(1,3,5))
```
