# Supplementary material for: Tigecycline-induced coagulation gene prognostic prediction model and intestinal flora signature in AML
Source: Front Immunol. 2024 Nov 14;15:1486592. doi: 10.3389/fimmu.2024.1486592 (PMC11602473; doi:10.3389/fimmu.2024.1486592)
Supplement: Supplementary file 13 [file DataSheet2.pdf]

```
dd <- datadist(rt)
options(datadist="dd")
f <- cph(Surv(futime, fustat) ~ riskScore+Age+Cytogeneticrisk, x=T, y=T, surv=T, data=rt, time.inc=1)
surv <- Survival(f)
#nomogram
nom <- nomogram(f, fun=list(function(x) surv(1, x), function(x) surv(3, x), function(x) surv(5, x),function(x) surv(7, x),function(x) surv(10, x),function(x) surv(12, x)),
                lp=F, funlabel=c("1-year survival", "3-year survival","5-year survival", "7-year survival", "10-year survival", "12-year survival"),
                maxscale=100,
                fun.at=c(0.99, 0.9, 0.8, 0.7, 0.5, 0.3,0.1,0.01))

#nomogram
pdf(file="Nomogram.pdf",height=7,width=8)
plot(nom)
dev.off()

#C-index
#riskScore+Cytogeneticwrisk+Age
library(Hmisc)
library(survival)
data <- read.table("calibration.txt")
f <- cph(Surv(futime,fustat)~ riskScore+Age+Cytogeneticrisk, x=T, y=T, surv=T,data=rt)
fp <- predict(f)
w=1-rcorrcens(Surv(futime,fustat) ~ predict(f), data = rt)
w

#riskScore
library(Hmisc)
library(survival)
data <- read.table("calibration.txt")
f <- cph(Surv(futime,fustat)~ riskScore ,x=T, y=T, surv=T,data=rt)
fp <- predict(f)
w=1-rcorrcens(Surv(futime,fustat) ~ predict(f), data = rt)
w

#Cytogeneticwrisk
library(Hmisc)
library(survival)
data <- read.table("calibration.txt")
f <- cph(Surv(futime,fustat)~ Cytogeneticrisk ,x=T, y=T, surv=T,data=rt)
```

```
fp <- predict(f)
w=1-rcorrcens(Surv(futime,fustat) ~ predict(f), data = rt)
w

#Age
library(Hmisc)
library(survival)
data <- read.table("calibration.txt")
f <- cph(Surv(futime,fustat)~ Age ,x=T, y=T, surv=T,data=rt)
fp <- predict(f)
w=1-rcorrcens(Surv(futime,fustat) ~ predict(f), data = rt)
w


#calibration curve
f1 <- cph(Surv(futime, fustat) ~ riskScore+RISS, x=T, y=T, surv=T, data=rt, time.inc=1)
cal1 <- calibrate(f1, cmethod="KM", method="boot", u=1, m=100, B=1000)
f1

f3 <- cph(Surv(futime, fustat) ~ riskScore+RISS , x=T, y=T, surv=T, data=rt, time.inc=3)
cal3 <- calibrate(f3, cmethod="KM", method="boot", u=3, m=100, B=1000)
f3

f5 <- cph(Surv(futime, fustat) ~ riskScore+RISS, x=T, y=T, surv=T, data=rt, time.inc=5)
cal5 <- calibrate(f5, cmethod="KM", method="boot", u=5, m=100, B=1000)
f5

f7 <- cph(Surv(futime, fustat) ~ riskScore+RISS, x=T, y=T, surv=T, data=rt, time.inc=7)
cal7 <- calibrate(f7, cmethod="KM", method="boot", u=7, m=100, B=1000)
f7

f10 <- cph(Surv(futime, fustat) ~ riskScore+RISS, x=T, y=T, surv=T, data=rt, time.inc=10)
cal10 <- calibrate(f10, cmethod="KM", method="boot", u=10, m=100, B=1000)
f10

f14 <- cph(Surv(futime, fustat) ~ riskScore+RISS, x=T, y=T, surv=T, data=rt, time.inc=14)
cal14 <- calibrate(f14, cmethod="KM", method="boot", u=14, m=100, B=1000)
f14
```
